# Effectiveness of Cinacalcet in Patients with Chronic Kidney Disease and Secondary Hyperparathyroidism Not Receiving Dialysis

**DOI:** 10.1371/journal.pone.0161527

**Published:** 2016-09-02

**Authors:** Ariadna Pérez-Ricart, Maria Galicia-Basart, Maria Alcalde-Rodrigo, Alfons Segarra-Medrano, Josep-Maria Suñé-Negre, José-Bruno Montoro-Ronsano

**Affiliations:** 1 Department of Pharmacy, Hospital Universitari Vall d’Hebron, Barcelona, Spain; 2 Department of Nephrology, Hospital Universitari Vall d’Hebron, Barcelona, Spain; 3 Pharmacy Faculty, Universitat de Barcelona, Barcelona, Spain; Hospital Universitario de la Princesa, SPAIN

## Abstract

**Background:**

Secondary hyperparathyroidism (SHPT) is a common complication in chronic kidney disease (CKD) patients. Cinacalcet could be a therapeutic option although its use is controversial in patients not receiving dialysis. Thus, the aim of this study is to assess the effectiveness and safety of cinacalcet in patients with CKD and SHPT without renal replacement treatment (RRT) and without renal transplantation (RT).

**Methods:**

A retrospective observational study was conducted. Patients were included if they had collected cinacalcet, under off-label use, during 2010 and 2011. Patients selected were followed from the beginning of cinacalcet therapy for one year of treatment.

**Results:**

A total of 37 patients were included with CKD stage 3 (38%), 4 (51%) and 5 (11%). Baseline mean PTH value was 400.86 ± 168.60 mg/dl. At 12 months, a 67% of patients achieved at least a 30% reduction in their PTH value (p<0.001; CI 49.7–83.6), and the overall mean reduction of PTH values was 38% (p< 0.001; IC -49.1, -27.5). A 28% of the patients achieved KDOQI PTH goals (p = 0.003, CI 12%-50%). At 12 months, mean serum calcium values decreased by 6% and mean serum phosphorus values increased by 13%. A 19% of patients experienced hypocalcemia episodes while an increase of 24% in hyperphosphatemia episodes was observed. A 25% of patients finished cinacalcet before a year of treatment. Main withdrawal reasons were: gastrointestinal and other discomfort (8%), hypocalcaemia (8%), non-compliance (3%), interactions (3%) and excess of efficacy (3%).

**Conclusions:**

Cinacalcet was effective in patients with CKD and SHPT not receiving dialysis. Electrolytic imbalances could be managed with administration of vitamin D and analogues or phosphate binders.

## Introduction

Secondary hyperparathyroidism (SHPT) is a complication of chronic kidney disease (CKD) that develops in the early stages and worsens as kidney function deteriorates. SHPT is caused by reduced phosphate excretion that leads to an increase of fibroblast growth factor-23, a reduced synthesis of 1,25-dihydroxy vitamin D and hypocalcemia, all of which promote parathyroid hormone (PTH) synthesis and release [1]. Then, PTH values then increase to restore calcium and phosphorus homeostasis. Persistent overproduction of PTH generates a systemic disorder characterized by high bone turnover and vascular calcification, which leads to an increased risk of bone fracture and cardiovascular mortality [2],[3].

The goal when treating SHPT is to reduce PTH values while maintaining calcium and phosphorus serum levels within the normal range [4]. Currently available treatment options include diet phosphorus restriction, vitamin D and analogues, phosphate binders, calcimimetics and, in severe cases, parathyroidectomy [1–4]. Cinacalcet hydrochloride (Amgen, Thousand Oaks, CA, United States) is a type II calcimimetic that increases the sensitivity to extracellular calcium of the calcium-sensing receptor (CaR) located in chief cells of parathyroid gland, thereby reducing PTH synthesis and release [4].

Currently, cinacalcet is indicated in the treatment of SHPT with end-stage renal disease undergoing maintenance with dialysis [5]. Since SHPT develops in the initials stages of CKD [2],[3], early management of mineral bone abnormalities is proposed in order to improve patient outcomes and quality of life [2]. Nevertheless, cinacalcet use in non-dialysis patients is highly controversial. In fact, in a phase III trial [6], cinacalcet reduced PTH values by 43% in non-dialysis patients versus placebo. However, it also produced hypocalcemia in 62% of patients, although it was mostly asymptomatic, and what is more worrying, an increase of 21.4% in phosphorus serum levels [6]. Although the efficacy of cinacalcet has been demonstrated, there is scarce data on its behavior in the clinical setting.

The aim of this study is to assess the effectiveness and safety of cinacalcet in clinical practice in patients with CKD and SHPT without renal replacement treatment (RRT) and without renal transplantation (RT).

## Materials and Methods

An analytic, retrospective, observational, non-placebo-controlled, single-center study was conducted. Adults diagnosed with CKD and SHPT but without RRT or RT were selected and included if they had collected cinacalcet, under off-label use, from the Outpatient Pharmacy Service (OPS) during 2010 and 2011, and had undergone at least a year of cinacalcet treatment before the beginning of RRT (hemodialysis or peritoneal dialysis) or RT. Patients were excluded if they were participating in an active cinacalcet clinical trial. Cinacalcet was prescribed in patients with elevated PTH value or sharp increase in PTH value. Patients had previously received standard treatment if calcium values allowed it. This study was presented and approved by Vall d’Hebron University Hospital Clinical Research Ethics Committee. Written or verbal consent to participate in this study was not systematically provided, since it was considered that the study was non-interventional and retrospective, collecting routine data from clinical records (one side); and that finding and contacting patients–most of them outpatiens- would be not feasible (other side). Thus, verbal consent was obtained when feasible (patients who underwent routine visit to hospital outpatient settings, during the study period). The Ethics Committee approved this procedure, as routinely proceeds with observational, retrospective studies.

The patients selected were followed from the beginning of cinacalcet therapy for one year of treatment, assessing medical data every 3 months. The data collected included biodemographic information, therapeutic data of cinacalcet, vitamin D and analogues, phosphorus binders and calcium supplements, and, analytic values of PTH, serum calcium and phosphorus levels, albumin, 25-OH-vitamin D, both serum and urine creatinine, proteinuria, alkaline phosphatase and gamma glutamil transferase. Patients were stratified by estimated glomerular filtration rate (eGFR) (CKD stage 3: 30–59 ml/min/1.73 m^2^, CKD stage 4: 15–29 ml/min/1.73 m^2^ and CKD stage 5: <15 ml/min/1.73 m^2^) [2] calculated by either the Cockroft-Gaul [7] or the Modification in Diet in Renal Disease [8] formula. Patients were also stratified by baseline PTH values. Serum PTH values were determined using a chemiluminescence immunoassay (Liaison XL). Cinacalcet dose was reported as average weekly dosage.

Effectiveness was defined as the proportion of patients with a 30% or greater reduction in PTH values from baseline at 12 months. Other effectivity outcomes were: proportion of patients who achieved National Kidney Foundation/Kidney Disease Outcomes Quality Initiative (NKF/K-DOQI) PTH goals of 70 pg/ml or less (CKD stage 3), 110 pg/ml or less (CKD stage 4) or 300 pg/ml or less4 (CKD stage 5) at 12 months^2^; and mean reduction in PTH value at 12 months. Moreover, the influence of vitamin D and analogues and baseline value of PTH were also evaluated. Safety was measured by calcium and phosphorus levels and number of hypocalcemia (two consecutive calcium values < 8.4 mg/dl) and hyperphosphatemia episodes (phosphorus > 4.5 mg/dl) [3]. Finally, the reasons for stopping treatment were recorded.

## Statistical Analysis

The principal analysis was conducted by intention to treat. To evaluate the effectiveness of primary and secondary variables, t-Student-Fisher test for continuous variables with normal distribution or a variance test such as two-way ANOVA for repeated measures was performed, considering time as a random factor and treatment as a fixed factor. If the variables did not follow a normal distribution, they were compared using the Friedman or Kruskal-Wallis test. Fisher’s exact test was applied for categorical variables. The number of patients was not previously defined, since all patients with available records from our OPS in 2010 and 2011 were considered for the study.

All the patients were included in safety analysis as all of them received at least one dose of cinacalcet. Adverse reaction events were tabulated by their incidence and severity. Adverse reactions were presumably related to the study treatment. Likewise, analytical parameters (serum calcium and phosphorus) were evaluated similarly to efficacy variables. Statistical analysis was conducted using SPSS V.15.0 (SPSS Inc, USA).

## Results

A total of 156 patients with CKD received cinacalcet from our OPS in 2010 and 2011. Exclusion criteria were: RT (83), primary hyperparathyroidism (13), SHPT treated with RRT or RRT initiated before less than a year of follow-up with cinacalcet (16), active cinacalcet trial (2) or clinical records not available (5). Finally, 37 patients were evaluated, whose baseline characteristics are summarized in Table 1. Creatinine, eGFR, albumin and calcium-phosphorus product values did not change significantly during the study (Table 2).

**Table 1:** Patient baseline caractheristics.

Characteristic	Cinacalcet; n = 37
***Sex*, *n (%)***	
Men	19 (51%)
Women	18 (49%)
***Age*, *mean (SD)*, *years***	65.32 ± 15.18 years
***CKD stage*, *n (%)*:**	
CKD 3	14 (38%)
CKD 4	19 (51%)
CKD 5	4 (11%)
***Cinacalcet dose*, *mean (SD)*, *mg/ week***	170 ± 86
***Concomitant treatment*, *n (%)***	
Vitamin D and analogues	19 (51%)
- Calcitriol	14 (38%)
- Paricalcitol	4 (11%)
- Calcifediol	1 (3%)
Phosphate binders	4 (11%)
- Calcium containing	4 (11%)
Calcium supplement use	1 (3%)
***Concomitant comorbidities*, *n (%)***	
Hypertension	34 (92%)
Dyslipemia	26 (70%)
Other cardiovascular disease	26 (70%)
Renal and urologic disease	22 (59%)
Diabetes	12 (32%)
Hyperuricemia	12 (32%)
Obesity	11 (30%)
Active smoker	8 (22%)
Active neoplasm	4 (11%)
Osteoporosis	3 (8%)
Endocrinology disease	3 (8%)
***Biochemical values*, *mean (SD)***	
PTH (pg/ml)	400.86 ± 168.60
Serum creatinine (mg/dl)	2.90 ± 1.17
Glomerular Filtration Rate	25.35 ± 11.09
Albumin (mg/dl)	4.20 ± 0.26
Serum calcium (mg/dl)	9.73 ± 0.70
Serum phosphorus (mg/dl)	3.81 ± 0.71
Calcium-phosphate product (mg^2^/dl^2^)	36.40 ± 6.72
25-hydroxy vitamin D (ng/ml)	14.34 ± 7.16

CKD: chronic kidney disease; PTH: parathyroid hormone; SD: standard deviation

**Table 2:** Evolution of biochemical values over the 12 months of treatment with cinacalcet (mean ± SD).

Biochemical value	Baseline	Month 3	Month 6	Month 9	Month 12
**PTH (pg/ml)**	400.86 ± 168.60	256.93 ± 110.71	278.75 ± 144.95	262.64 ± 133.96	224.31 ± 132.88
**sCr (mg/dl)**	2.90 ± 1.17	2.96 ± 1.34	2.90 ± 1.42	2.93 ± 1.31	3,15 ± 1.57
**GFR**	25.35 ± 11.09	23.02 ± 10.15	25.46 ± 13.36	24.18 ± 11.24	27.97 ± 35.28
**Alb (mg/dl)**	4.20 ± 0.26	4.06 ± 0.82	4.18 ± 0.27	4.21 ± 0.31	4.17 ± 0.25
**sCa (mg/dl)**	9.73 ± 0.70	9.31 ± 0.77	9.31 ± 0.61	9.00 ± 1.27	9.18 ± 0.79
**sP (mg/dl)**	3.81 ± 0.71	4.05 ± 1.04	4.05 ± 0.81	4.24 ± 0.88	4.32 ± 1.05
**CaxP (mg**^**2**^**/dl**^**2**^**)**	36.40 ± 6.72	38.21 ± 11.32	36.24 ± 7.55	37.17 ± 8.70	37.44 ± 9.75
**25-OHD (ng/ml)**	14.34 ± 7.16	14.18 ± 6.89	19.21 ± 13.25	22.45 ± 19.14	17.88 ± 9.60

sCr: serum creatinine; GFR: glomerular filtration rate; Alb: albumin; sCa: serum calcium; sP: serum phosphorus; CaxP: calcium-phosphate product; 25-OHD: 25-hydroxy vitamin D

At the end of the study, the mean cinacalcet dose was 168 ± 131 mg/week (24 mg/day). The most frequent cinacalcet daily dose was < 30 mg/day (43%) and 30 mg/day (30%), followed by 30–60 mg/day (17%), 60 mg/day (7%) and >60 mg/day (3%). For patients with < 30 mg/day, most frequent prescription was 30 mg four times a week (13%), followed by 30 mg five times a week (10%) and 30 mg three times a week (10%), 30 mg six times a week (7%) and 30 mg weekly (3%). Individualized adjustments were made when needed, according to the biochemical values. At baseline, 51% and 11% of patients used vitamin D and analogues and phosphate binders respectively, increasing up to 75% and 28% respectively at the end of the study. Only 3% of patients used calcium supplements. At the end of the study, the vitamin D and analogues used were calcitriol (43%), paricalcitol (27%) and calcifediol (8%), while the phosphate binders were calcium-containing (30%), lanthanum carbonate (5%) and sevelamer (3%).

**Fig 1:**
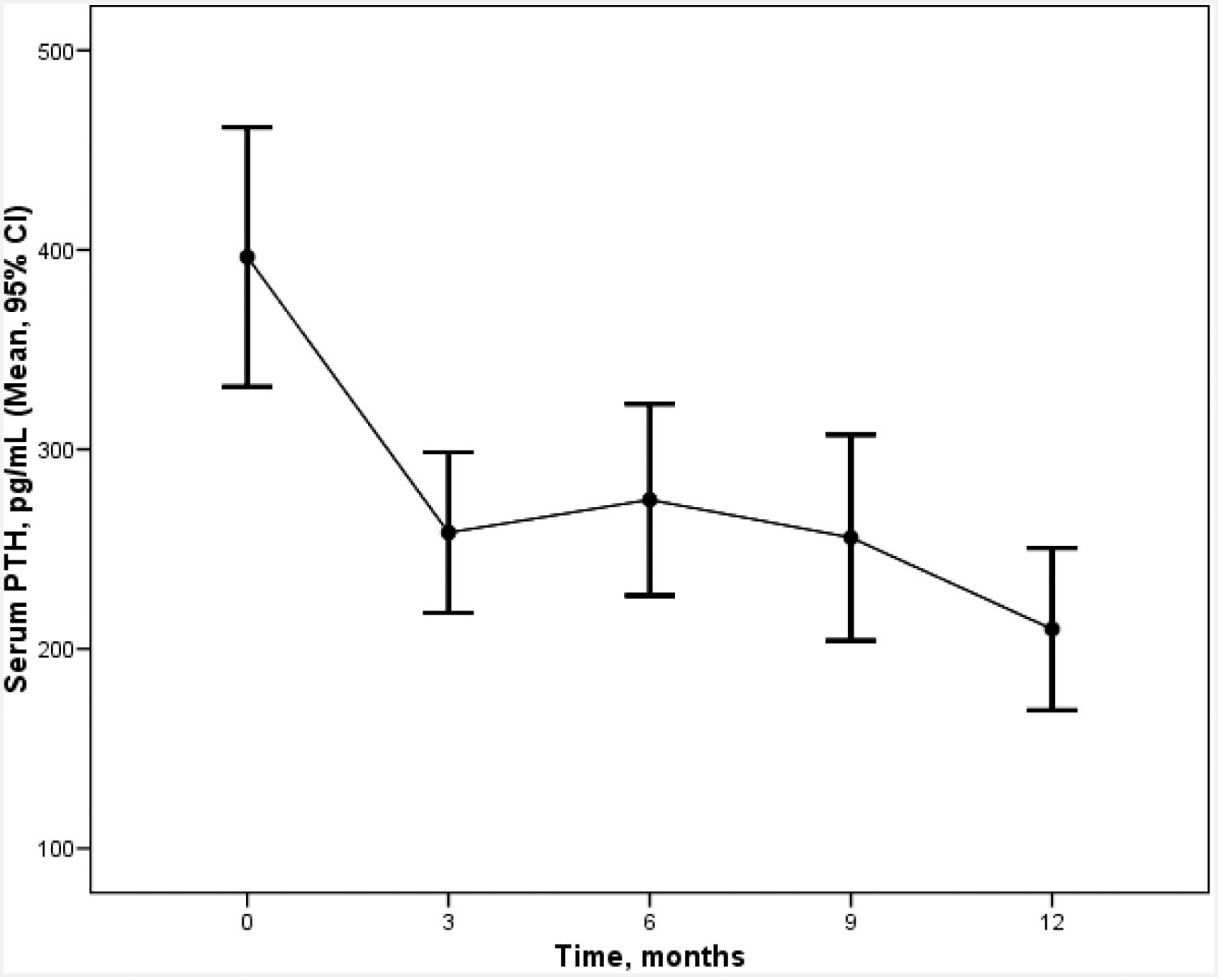
Evolution of PTH values over the 12 months of treatment with cinacalcet (mean ± SD).

As shown in Table 3, cinacalcet significantly reduced PTH values, even considering only the first 3 months. The evolution of PTH values over the 12 months of treatment is shown in Fig 1. Better results were obtained with basal PTH values > 300 pg/ml (Table 3). Cinacalcet treatment also led to 28% of the patients achieving NKF/K-DOQI PTH goals (p = 0.003, CI 12%-50%). No significant differences were observed in effectiveness outcomes between CKD groups (Table 4) except for the achievement of NKF/K-DOQI PTH goals.

**Table 3:** Main effectiveness outcomes after 3 and 12 months with cinacalcet therapy.

	*Proportion of patients with a reduction of PTH ≥ 30%*	*Mean PTH reduction*
**Overall results of the study**		
**3 months**	53% p<0.001 (CI: 35.3–70.6)	-27% p< 0.001 (CI: 40.0–14.1)
**12 months**	67% p<0.001 (CI: 49.7–83.7)	-38% p< 0.001 (CI: 49.1–27.5)
**Results according basal PTH**		
**3 months**	PTH>300: 73%	PTH>300: -44%
	PTH<300: 17%	PTH<300: +3%
**12 months**	PTH>300: 71%	PTH>300: -42%
	PTH<300: 58%	PTH<300: -32%

PTH: parathyroid hormone; CI: confidence interval

**Table 4:** Effectiveness outcomes at 12 months of cinacalcet therapy according CKD stages.

Outcome	CKD3	CKD4	CKD5	p-value
Proportion of patients with a reduction of PTH ≥ 30%*	64%	63%	100%	p = 0.461
Mean PTH reduction*	-35%	-36%	-70%	p = 0.157
Proportion of patients with NKF/K-DOQI PTH goal achievement**	0%	17%	86%	p = 0.001

CKD: chronic kidney disease; PTH: parathyroid hormone; KDOQI: kidney disease outcomes quality initiative

*CKD at baseline

**CKD at 12 months

No significant changes in a reduction of PTH values ≥ 30% were observed after comparing patients treated with vitamin D and analogues for longer or shorter than 6 months (p = 0.22).

**Fig 2:**
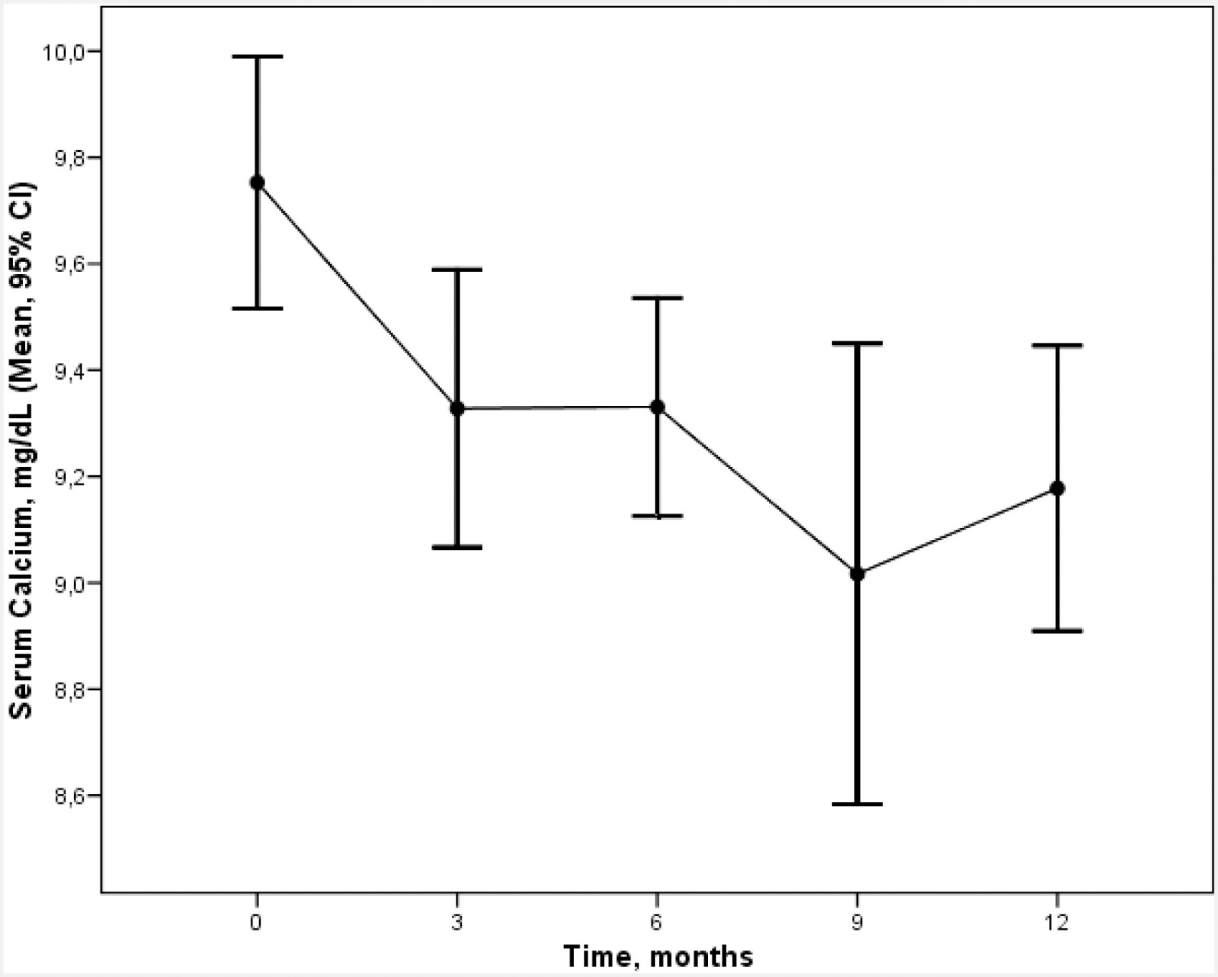
Evolution of calcium values over the 12 months of treatment with cinacalcet (mean ± SD).

**Fig 3:**
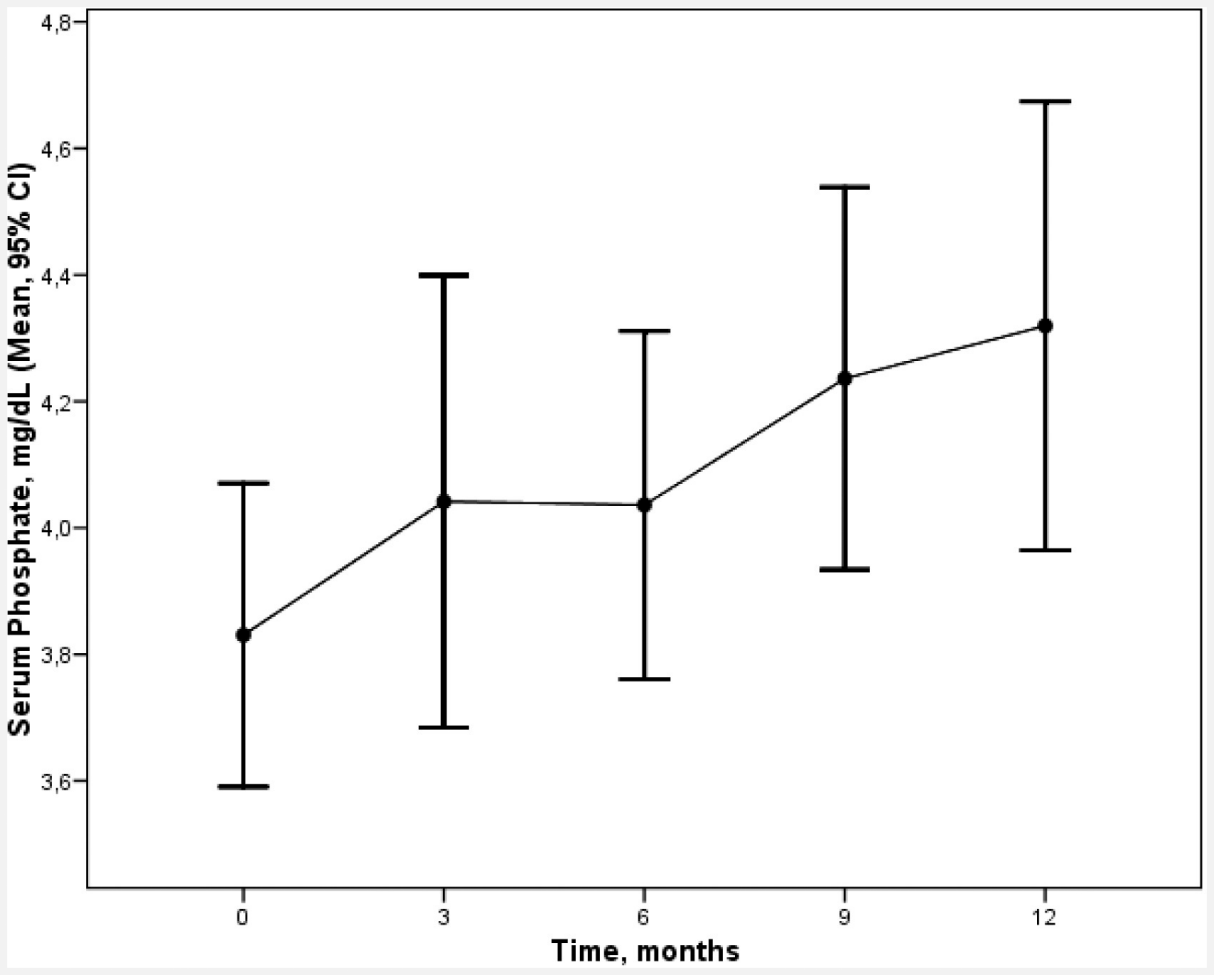
Evolution of phosphate values over the 12 months of treatment with cinacalcet (mean ± SD).

Cinacalcet reduced calcium values and increased phosphorus values, as can be seen in Figs 2 and 3. At baseline, the mean calcium value was 9.7 ± 0.7 mg/dl, which was reduced by 4% and 6% at 3 and 12 months respectively. At baseline, the mean phosphorus value was 3.8 ± 0.7 mg/dl, which increased by 6% and 13% at 3 and 12 months respectively.

Cinacalcet treatment produced hypocalcemia and hyperphosphatemia. At baseline, no patients presented hypocalcemia, while at the end of the study, 19% of patients did. Hypocalcemia was greater in patients with CKD 5 (50%) followed by patients with CKD 3 (21%) and CKD 4 (11%). At the end of the study, patients with hypocalcemia showed a mean calcium value of 7.87 +/- 0.25 mg/dL. No patients reported calcium value less than 7.5 mg/dl. Mean duration of hypocalcemia episodes was 6 ± 3.3 months. Corrective measures consisted in decreasing or stopping cinacalcet and/or increasing vitamin D and analogues. At baseline, 14% of patients presented hyperphosphatemia, after cinacalcet treatment, this value increased to 38%. Hyperphosphatemia was more prevalent in patients with CKD 4 (initial 5%; final 42%) and CKD 3 (initial 7%; final 21%), while no change was observed in patients with CKD 5 (initial 75%; final 75%).

A 25% of patients discontinued cinacalcet treatment before a year, although 5% of these restarted. Cinacalcet withdrawal reasons were: gastrointestinal discomfort and other intolerances (8%), hypocalcemia (8%), non-compliance (3%), interactions (3%) and excess of effectiveness (3%). In addition, 10% of patients experienced adverse events that did not make them discontinue the treatment with cinacalcet: 2 patients, gastrointestinal discomfort, 1 patient, muscle spasm, 1 patient, paresthesia and 1 patient, alopecia.

## Discussion

Cinacalcet is usually used in patients with CKD and SHPT undergoing maintenance with dialysis, but patients not on dialysis could also benefit. In this study, a 67% of patients achieved at least a 30% reduction in their PTH value, and the overall mean reduction of PTH values was 38%. These results are consistent with clinical trials [6],[9], although they are lower than those of other observational studies [10–13]. Differences could be explained by the type of patient evaluated, PTH baseline values, cinacalcet dose and the patients’ co-morbidities. Thus, Chonchol et al [6] evaluated patients with less severe kidney disease, lower baseline PTH values and receiving higher doses of cinacalcet. However, other observational trials evaluated patients with more advanced CKD whose cinacalcet doses were slightly higher [10–13].

In addition, in our study, cinacalcet effectiveness was already significant in the first three months. Thus, a 53% of patients achieved a reduction of 30% or greater in the first 3 months of treatment. An important reduction of PTH values in the first three months with a relatively low dose of cinacalcet is considered a good prognostic factor of the cinacalcet response [14]. Moreover, the *National Institute for Clinical Excellence* recommended continuing cinacalcet in hemodialysis patients only if patients achieved at least a 30% reduction in their PTH value in the first four months [15].

From our results, it could be argued that the effectiveness of cinacalcet is conditioned by baseline PTH values regardless of CKD stage, which would support the use of cinacalcet in patients in the early stages of CKD with elevated PTH levels. Similarly to Chonchol et al [6], no differences were observed in the main endpoint across CKD stages. However, in this study, 71% of patients with baseline PTH values > 300 pg/ml achieved at least a 30% reduction in their PTH value at 12 months, with a mean reduction of 42%. Nevertheless, the influence of PTH baseline values on the efficacy of cinacalcet is controversial in hemodialysis studies [14],[16–25]. In clinical practice trials in non-dialysis patients, higher baseline PTH values correlate to greater reduction, although no linear relation is observed [10–13].

Another controversial issue is the influence of the concomitant treatment with vitamin D and analogues on cinacalcet results, as vitamin D may also regulate PTH synthesis and release [1–3]. In this study, no differences were observed between patients treated for longer or shorter than six months with vitamin D and analogues in the main endpoint. These results were similar to those of other studies in non-dialysis and hemodialysis patients [6],[18–20]. Consequently, it seems that cinacalcet has an important intrinsic effect on reducing PTH values. Moreover, cinacalcet treatment reduces doses of vitamin D and analogues used in some studies [19],[21],[25].

Although cinacalcet was shown to be effective, only 28% of our patients achieved the PTH goals recommended by the NKF/K-DOQI guideline [2]. Success was low for patients with CKD 3 and CKD 4 and high only for patients with CKD 5. Low rates of achieving the goals were also observed in other non-dialysis trials [26], while higher compliance was observed in hemodialysis patients [14],[17–25]. Difficulties in clinical management or patient adherence to treatment could partially explain this low compliance [26]. Nevertheless, the KDOQI PTH targets for patients with CKD 3 and 4 have been questioned as they are based on expert opinion [2],[26]. PTH is known to be correlated with high-turnover bone disorder but there is a lack of association trials between clinical outcomes and PTH levels in non-dialysis patients [3]. Consequently, the issue of an optimal PTH level remains unsolved in this group of patients [3].

In hemodialysis patients, the efficacy of cinacalcet was comparable to that in non-dialysis patients, although with higher cinacalcet doses and higher baseline PTH values [14],[17–25]. However, the electrolyte profile is different. In this study, cinacalcet treatment reduced calcium values and increased phosphorus values, as also found in Chonchol et al [6]. In contrast, in hemodialysis patients, cinacalcet reduced calcium values but maintained or decreased phosphorus values [14],[17–25].

Cinacalcet-related hypocalcemia is widely reported, in both dialysis and non-dialysis patients [6],[9–11],[14],[17–25]. Hypocalcemia seems to be related to a reduction of PTH values, which in turn, decreases the release of calcium from bone [4]. However, calcium imbalance remains the most controversial issue concerning cinacalcet treatment in non-dialysis patients. In Chonchol et al [6], 62% of patients were observed to experience hypocalcemia episodes, although they were mostly asymptomatic [6]. Consequently, the regulatory authorities denied approval of cinacalcet for non-dialysis patients [5]. In our study, a 19% of patients underwent hypocalcemia episodes, and they led to suspension of cinacalcet treatment in a 8% of cases. In contrast, clinical practice trials showed high variability, with hypocalcemia reported in from 8% to 70% of patients [10–13]. These variations could be explained by differences in vitamin D and analogues and phosphate binder administration. For instance, in our study, a greater proportion of vitamin D and analogues and a lower proportion of phosphate binders were used than in Chonchol et al [6]. Patient adherence to concomitant therapy could also be an important issue.

Another worrying issue is the increase in phosphorus values. In CKD 3 and CKD 4 patients, residual renal function remains, and this could produce an increase in phosphorus tubular re-absorption as a consequence of decreased PTH value [10],[27]. The DOPPS study considered that values of PTH > 600 pg/ml, Ca > 10 mg/dl and P > 7 mg/dl were related to an increased risk of all-cause mortality [28]. Palmer et al [29], however, only elevated phosphorus was found to increase mortality risk in CKD patients with and without RRT. In fact, all-cause mortality risk increased by 18% for each increase of 1 mg/dl in phosphorus levels (RR 1.29; 95% 1.12–1.48) in non-dialysis patients, with a more consistent relationship when phosphorus levels were > 5.5 mg/dl [29]. In addition, elevated phosphorus values are considered to be the primary cause of vascular calcification [30]. In this study, 24% of patients experienced hyperphosphatemia episodes. This value was similar to that found by Chonchol et al [6], but it could not be compared with other observational trials because this study is the first to consider it. The sparse use of phosphate binders and the extensive use of calcitriol in our study could partially explain these results.

Consequently, some authors believe that cinacalcet should not be used in non-dialysis patients and other alternatives are preferred [31]. Nevertheless, standard therapies with vitamin D and phosphate binders are not risk free. Vitamin D stimulates intestinal absorption of calcium and phosphorus, increasing both hypercalcemia and hyperphosphatemia episodes and vascular calcification [1]. Calcium-based phosphate binders could increase both calcium and calcium-phosphorus product levels [1]. In contrast, paricalcitol has showed similar efficacy with a slightly better adverse events profile than cinacalcet [32]. However, no comparative effectiveness trial of paricalcitol and cinacalcet has been conducted.

This work has several limitations. It is an observational retrospective single-center trial. Consequently, relevant data could be lost and bias could be introduced. However, effectiveness in clinical practice can be meaningfully assessed. In addition, few patients could be followed, although this study is the largest reported in the clinical setting. Moreover, only biochemical surrogate parameters, and not hard outcomes, could be evaluated.

To sum up, this study suggests that cinacalcet treatment could be a valid option for non-dialysis patients and it shows effectivity after three months of treatment. Moreover, the effectiveness of cinacalcet seems to be conditioned by baseline PTH values, regardless CKD stage. However, calcium and phosphorus should be monitored to avoid hypocalcemia and hyperphosphatemia.Hypocalcemia could be managed with administration of vitamin D and analogues. Nevertheless, cinacalcet treatment was associated with hyperphosphatemia in a high proportion of patients. Prospective studies are needed to study the impact on cardiovascular risk and/or mortality in these patients.
